# Effect of Iranian Propolis on Salivary Total Antioxidant Capacity in Gamma-irradiated Rats

**DOI:** 10.5681/joddd.2014.042

**Published:** 2014-12-03

**Authors:** Sara Aghel, Mahdi Pouramir, Ali Akbar Moghadamnia, Dariush Moslemi, Tahere Molania, Leila Ghassemi, Mina Motallebnejad

**Affiliations:** ^1^Assistant Professor, Department of Oral Medicine, Faculty of Dentistry, Babol University of Medical Sciences, Babol, Iran; ^2^Professor, Department of Biochemistry, Babol University of Medical Sciences, Babol, Iran; ^3^Professor, Department of Pharmacology, Babol University of Medical Sciences, Babol, Iran; ^4^Assistant Professor, Department of Radiation Oncology, Babol University of Medical Sciences, Babol, Iran; ^5^Assistant Professor, Faculty of Dentistry, Mazandaran University of Medical Sciences, Babol, Iran; ^6^General Dental Practitioner; ^7^Associate Professor, Dental Materials Research Center, Faculty of Dentistry, Babol University of Medical Sciences, Babol, Iran

**Keywords:** Antioxidant activity, propolis, saliva, radiation therapy

## Abstract

***Background and aims.*** The antioxidant and anti-inflammatory properties of propolis were studied. Since saliva containsantioxidants and radiotherapy of the head and neck mainly affects the saliva, salivary antioxidant defensive mechanism iscompromised with oxidative stress produced by radiation therapy. Therefore, the aim of the present study was to investigatethe effect of propolis on salivary total antioxidant capacity in irradiated rats.

***Materials and methods.*** The study was conducted on 28 rats, 7-11 weeks of age (160±20 g), divided into four groups:saline with no radiation (S), saline and radiation (SR), propolis with no radiation (P) [400 mg/kg IP], propolis and radiation(PR) [400 mg/kg IP]. SP and PR were exposed to 15 Gy of gamma irradiation for 7 minutes and 39 seconds. The rats received intraperitoneal injections each day for 10 days, and their tongues and lips were daily examined for mucositis; salivasample were also taken three times on days 0, 6, and 10.

***Results.*** Mucositis incidence appeared to be delayed in the PR compared to the SR, and the severity was significantlyhigher in the SR compared to the PR. No significant alterations were observed in salivary antioxidant levels during the ex-periment, except the SR group in which a significant reduction was found.

***Conclusion.*** Propolis might reduce and delay radiation-induced mucositis in animal models; it might be able to prevent thereduction in salivary antioxidant levels in irradiated rats as well.

## Introduction

Mucositis is caused by early effects of radiation on rapidly dividing mucosal basal cells due to the effect of radiation on DNA replication and proliferation of mucosal cells, leading to a decrease in basal epithelial regeneration and eventually mucosal atrophy, collagen breakdown and ulceration.^1,2^

Radiation destroys the cells and creates charged molecules which, per se, interfere with biochemical processes in the cells via direct damage to DNA and production of free radicals.^3^ Oxidative stress, resulting from an imbalance between free radicals and antioxidant defense system, contributes to cellular damage in the oral cavity.^4^

Antioxidants are present in all the body tissues and fluids, including the saliva. Saliva is the first biological fluid containing free radicals of consumed food.^4,5^ Salivary antioxidant system consists of numerous molecules, among which uric acid and peroxidase systems are the most common. Glutathione and superoxide antioxidant enzymes inside the cell and low molecular weight antioxidants such as ascorbic acid, α-tocopherol and β-carotene in extracellular fluid have protective effects on the cell. Antioxidant activity of body fluids and plasma, such as those covering the lung epithelial surface, have been widely studied; however, saliva-associated research is limited.^5^ Given the fact that damage to the salivary glands is the major and devastating complication of radiotherapy,^6^ the alteration in the antioxidant capacity of saliva and its protective effect can be expected as a result.

Propolis has been of growing importance in recent years. Flavonoids and other phenolic compounds are the significant active components in propolis with notable pharmacological and biological effects, such as antioxidant, antimicrobial, anti-inflammatory, anti-tumor traits.^[Bibr R04],7-15^, Recently, several laboratory and animal studies have been undertaken in line with protective effects of propolis against radiation mucositis.^12,16-19^, Moreover, clinical and histopathologic properties of propolis have been shown on irradiated oral mucosa in some investigations.^13-15^Recently, the effect of propolis has been demonstrated on the reduction of oral mucositis symptoms in irradiated rats and the antioxidant, antibacterial and immunoregulatory properties of propolis have also been pointed out in reducing the mucosal damage. Antioxidant property of propolis in the serum has been indicated in this study as well.^13^ In an in vitro study, antioxidant and protective properties of propolis have been noted against oxidative stress in human saliva.^4^ Recently two separate studies were carried out by the authors, which showed clinical effects of propolis on radiation-induced mucositis in an animal model.^14,15^ Since propolis was proposed to have many properties such as antioxidant, antibacterial, antifungal, radioprotective and antitumor effects, the present study was designed to evaluate salivary antioxidant effects of propolis during clinical course of radiation-induced mucositis in order to interpret the mechanism of its clinical effect on mucositis.

## Materials and Methods

The present study was an in vitro study which was carried out from January to February 2011 in the Animal Care Center of Babol University of Medical Sciences. The project was approved by the Research Council as well as the Research Ethics Committee of the University.

### Animals

The study was performed on 28 male Wistar rats, aged 7-11 weeks and weighing 160±20 g. After two weeks of acclimatization, the rats were housed in metal laboratory cages under standard conditions (temperature: 22±2°C, dark/light cycles: 12/12 hours) with access to food and water ad libitum.^15^

### Propolis 

Fresh propolis, produced in some regions of the province, was acquired from Apiculture Department of Faculty of Applied Science and Technology. To prepare aqueous solution of propolis, 50 g of propolis was added to the water solution in a beaker, and Tween 80 was used as a dispersing agent for better homogenization with propolis. For this purpose, a homogeneous mixture was first made by adding the pre-weighed amount of propolis in 10 mL of Tween 80, to which distilled water, 70°C, was then gradually added until a homogenized mixture was obtained. At the same time, the mixture was stirred by the magnet on hot plate magnetic stirrer for 6 hours at 50-60°C. Regarding the gradual precipitation of propolis, each session the mixture was sufficiently stirred for 10 minutes before intraperitoneal injection of aquatic solution of propolis.

### Study Procedures

A pilot study was conducted to observe the process of radiation, onset time of radiation-induced mucositis, the most important radiation effects (sixth day) and the endpoint of the experiment (tenth day). The rats were randomly divided into four groups of seven rats each as follows: the group receiving saline (S), the group receiving saline and gamma radiation (SR), the group receiving 400 mg/kg of propolis (P), and the group receiving 400 mg/kg of propolis and gamma radiation (PR). At the beginning of the study, all the groups were marked and weighed prior to radiation. Propolis, 400 mg/kg, was then injected intraperitoneally (IP) to P and PR group rats and saline was injected to S and SR group rats as the first injection on the morning of radiation day which continued for 10 consecutive days. Saliva sampling was performed in 3 stages: on days 0, 6 (maximum severity of mucositis) and 10 (end of the experiment). To obtain saliva samples, the rats were partially anesthetized with midazolam (25 mg/kg), and pilocarpine (0.5 mg/kg) was then injected intraperitoneally to stimulate salivary flow. Saliva samples were kept in the freezer at -20°Cuntil salivary analysis. After the injections and saliva sampling, SR and PR group rats were transferred to hospital for irradiation. Prior to irradiation, the rats were anesthetized by ketamine (100 mg/kg IP). The rats were completely immobilized on a special shield and exposed to gamma radiation (Teraton 780, Canada) with ^60^Co. The device was operated at 1.25 million electron volts energy at a dose rate of 15 gray for 7 minutes and 39 seconds. The tube placement was set in a way that the rats' whole cranium was in the field. At the end of radiation, the rats were returned to the Animal Care Center and their tongues and lips were examined for the incidence of mucositis during the 10-day period of the experiment using Parkin's clinical scale as follows: scale 0, normal; scale 0.5, slightly pink; scale 1, slightly red; scale 2, extremely red; scale 3, local desquamation; scale 4, exudation less than one-half of the lip; scale 5, exudation more than one-half of the lip.^20^

The examiner was blind to the experiment. The first examination was performed 24 hours after irradiation. Injection and examination continued up to the tenth day (according to the pilot study). On the 10th day after saliva sampling, the rats were sacrificed after anesthesia. Three saliva samples prepared were transported to the Biochemistry Laboratory and underwent centrifugation at 3000 rpm for 15 minutes and transferred to the test tubes after deposition of impurities. Using FRAP (Ferric reducing antioxidant power) technique the total antioxidant activity was calculated.^21^ This method is based on the reduction of Fe^3+^ to Fe^2+^ in the presence of antioxidants. FRAP reagent contains TPTZ (2, 4, 6-tripyridyl-s-triazine; sigma), 10 mmol/L in 40 mmol/L of HCL, plus FeCl_3_,20 mmol/L, and buffer acetate, 0.3 mol/L (PH: 3.6), in the ratio of 10:1:1. The reagent was freshly prepared and heated for 5 minutes at 37°C. The working FRAP reagent (1.5 mL) was mixed with 50 μL of serum. Just after 10 minutes at 37°C, the absorption was read at 593 nm and compared with the standard. The standard solution was FeSO_4_ (125, 250, 500, and 100 µmol/L), based on which the standard curve was drawn.^21^

### Statistical Analysis

Data are presented as mean (±SD) in tables and figures. Mucositis-related data were also analyzed by one-way ANOVA. To compare the severity of mucositis between the study groups, Mann-Whitney test was used between each two groups. In addition, paired t-test was used for the comparison of antioxidant variables between the study groups. P<0.05 was considered significant.

## Results

The study was performed on 28 Wistar rats divided into four groups of seven rats each.

### Mucositis

**Figure 1.**
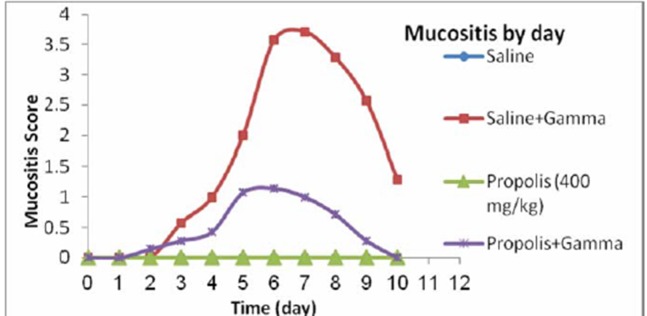
The grade of mucositis in the PR and SR groups during the 10 days of experiment.

Figure 1 shows the process of mucositis in both SR and PR groups. The mean mucositis onset time was 2.43±0.8 days in the SR and 5.2±2.7 days in the PR group. As shown, mucositis appeared significantly later in the PR than the SR group (P= 0.025, t-test). The severity of mucositis was higher in the SR compared to the PR group on day 10, and the difference was significant on the fourth to the ninth days of the experiment (P = 0.017, P = 0.038, P = 0.011 and P = 0.022, respectively).

### Antioxidants

Salivary antioxidant levels are presented in Table 1. No significant alteration was observed in the S, P and PR groups after the 10-day experiment; however, salivary antioxidant concentration significantly decreased after ten days in the SR group (P=0.041).

**Table 1:** Salivary antioxidant levels in the S, SR, P and PR groups at three intervals during the 10-day study period

Groups	S	SR	P	PR
Day 0	348.86 ± 151.29	344.00 ± 187.40	414.00 ± 213.09	427.25 ± 364.60
Day 6	345.71 ± 67.7	527.50 ± 194.51	423.93 ± 242.14	493.57 ± 90.76
Day 10	244.38 ± 62.90	205.28 ± 114.42	238.44 ± 156.97	320.94 ± 282.20

## Discussion

In the present in vitro study, the effect of propolis on total salivary antioxidant capacity was investigated in gamma-irradiated rats. According to radiation-induced oxidative-stress and anti-inflammatory, antioxidant and antimicrobial properties of propolis, it is likely that antioxidant property of the propolis will lead to a reduction in the severity of mucositis.^7-15^ In vitro studies by Motallebnejad^14^ and Ghassemi,^15^ similar to the present study on rats, showed low-intensity and delayed incidence of mucositis in propolis-receiving groups during the 10-day period of the study. The clinical results of the present study well confirmed the above findings. In an investigation by Benderli et al,^13^ radiation increased the serum MDA (Malondyaldehide) and decreased the activity of catalase and superoxide dismutase (SOD); higher levels of SOD and catalase were also measured in the serum of propolis-receiving group, indicating the beneficial effect of propolis on the serum antioxidant activity. Since mucosal histopathological examination was the aim of the present study, the study period was longer compared to previous research studies;^13-15^ hence, saliva samples were taken from the animals after improvement of oral lesions (ten days) for the evaluation of antioxidant changes, and it was observed that increases in salivary antioxidants were not parallel to improvements in oral mucositis lesions. Unfortunately, due to lack of serum samples in this study, no judgment can be made on serum changes, like what was seen in Benderli study, and merely the salivary antioxidant changes can be judged. Since salivary antioxidant alterations were observed ten days after irradiation rather than on the sixth day (the maximum mucositis), saliva antioxidant changes therefore appeared later than clinical appearance of mucositis.

Meanwhile, lack of reduction in the saliva antioxidant levels in the PR group demonstrates effectiveness of propolis in preventing the decrease in antioxidant concentration, and because these alterations were found at the end of the study period, evaluation of the effects of propolis on salivary antioxidants following radiation requires further investigation with longer durations (more than ten days).

Despite the clinical effect of propolis on early-onset symptoms of radiation (mucositis), antioxidant effects on saliva were not parallel with the process of mucositis; in this context, two points are noteworthy to be recommended: first, antioxidant effect of propolis is likely to be parallel with the process of mucositis through the serum, and simultaneous evaluation of the serum and saliva is thus needed; second, antioxidant effect of propolis does not probably prevent the occurrence of mucositis and it affects mucositis through another mechanism. In the end, it is suggested that further studies be conducted to investigate antioxidant effects of propolis on saliva and serum simultaneously and in longer time periods as well as on other mechanisms of propolis effect on radiation-induced mucositis.
